# Identification of human immunodeficiency virus -1 E protein-targeting lead compounds by pharmacophore based screening

**DOI:** 10.15537/smj.2022.43.12.20220599

**Published:** 2022-12

**Authors:** Mazen M. Almehmadi, Alaa A. Shafie, Mamdouh Allahyani, Tahir Muhammad, Soukayna Baammi, Abdulelah Aljuaid, Abdulraheem A. Almalki, Ahad Amer Alsaiari, Amal Adnan Ashour

**Affiliations:** *From the Department of Clinical Laboratory Sciences (Almehmadi, Shafie, Allahyani, Aljuaid, Almalki, Alsaiari), College of Applied Medical Sciences, and from the Department of Oral (Ashour), Maxillofacial Surgery and Diagnostic Sciences, Faculty of Dentistry, Taif University, Taif, Kingdom of Saudi Arabia; from the Molecular Neuropsychiatry & Development (MiND) Lab (Muhammad), Campbell Family Mental Health Research Institute, Centre for Addiction and Mental Health, Toronto, Canada; and from the African Genome Centre (Baammi), Mohammad VI Polytechnic University, Benguerir, Morocco.*

**Keywords:** HIV, protein E, vaccination, immunology

## Abstract

**Objectives:**

To identify potential compounds by seeking the knowledge of molecular interactions between human immunodeficiency virus (HIV) glycoprotein (gp) 120 protein and anti-HIV drug (BMS-488043).

**Methods:**

This study is a computational structure-based drug design study, carried out at University of Taif, Saudi Arabia and African Genome Centre (AGC), Mohammed VI Polytechnic University, Benguerir, Morocco from January 2021 to March 2022. Initially, using the docked structure of gp120 with BMS-488043, a structure-based pharmacophore model was created. The generated model was utilized for virtual screening of the ZINC and ChemBridge database in order to identify hit compounds. To further assess the time-dependent stability of the selected complexes, computer simulation was performed.

**Results:**

From pharmacophore-based screening, 356 hits were obtained from both the database. The docking studies of the retrieved hit compounds reveal that all the compounds fit into the binding site of gp120. However, based on the significant interactions with the crucial residues and docking scores four compounds were suggested as potential hits. MD simulation of ChemBridge14695864 and ZINC06893293 in complex with gp120 suggested that both compounds significantly stabilized the receptor.

**Conclusion:**

These findings could aid in the design of effective drugs against HIV by inhibiting the interaction between gp120 and CD4.

**T**he human immunodeficiency virus (HIV), also known as HIV, is the pathogen that ultimately results in acquired immune deficiency syndrome (AIDS). AIDS, the most epidemic disease, was first discovered in 1981.^1^ The pandemic of HIV/AIDS imposes the rapid development of novel anti-HIV therapies and preventative vaccines.^2^ HIV replication consists of an initial “entry” phase followed by a “post-entry” phase.^3^ The post-entry phase is the most chronic phase because-different steps such as integration, reverse transcription, protein synthesis, and protein packaging occurred. In the past decades, different HAART (highly active antiretroviral therapy) antiviral drugs have been used to reduce AIDS. These medications include a variety of protease and RNA-dependent DNA polymerase inhibitors, which block the HIV-1 post-entry pathway.^4,5^ However, resistant against these viral inhibitors/blockers have been reported.^6,7^ Recently in a study, HAART was not succeeded in effectively curing the infected patients of HIV-1.

A prevention cures plan against HIV-1 viral infection is in the viral entry phase for the new drug development. Glycoprotein is a therapeutic target and has a significant role in receptor binding and the study of HIV infection.^8,9^ Human immunodeficiency virus’ attack strategy is complex and involves multiple steps, including attachment to the host receptor and co-receptors and subsequent membrane fusion. Glycoprotein (gp) 120 forms the outer spikes which are sticky with the HIV particle and its molecular weight is 120 kilodaltons as its name indicates. Alongside the glycoprotein gp41, it is found on the surface of the HIV virus in a trimeric state.^10^ The most important function of gp120 is to attach with human CD4 cells.^11^ The gp120 consists of 5 α-helices, 25 β-strands, and 10 defined loop segments and also has chemically determined Disulphide-bridges. The gp120 is a polypeptide chain folded into the 2 main domains, and also certain excursions originate from this body. According to molecular weight, gp120 has carbohydrate side chains to avoid immune recognition.

The exterior gp120 and transmembrane protein gp41, viral envelope proteins regulate these multistep processes. Glycoprotein41 and gp120 both are formed from gp160 precursors and projected from the vision’s membrane. Identification of drugs that obstruct the virus’s entry into the host cell’s machinery represents a promising avenue for discovering the effective anti-HIV therapy. The interaction of gp120 with the human CD4 cell surface receptor initiates HIV-1 infection. When gp120 binds to CD4 receptors and co-receptors like CXCR4 or CCR5, a cascade of conformational changes occurs in both gp41 and gp120, allowing the gp41 fusion peptide to enter the human cell membrane and ultimately resulting in the fusion of the host cell membrane and the virus.^12-16^ Each and every step of the aforementioned process is considered as a potential target for blocking HIV-1 entry into the host cell’s genome. Recently, BMS-378806 along with a number of other similar compounds were discovered as highly potent and possible HIV entrance inhibitors.^17-25^ Among these explored compounds the BMS-488043 is an indole ring derivative, it has excellent pharmacokinetic activity as compared to BMS-378806.^26^

This study aim to developed a valid pharmacophore model based on the binding interactions between the known inhibitors and HIV-1 envelop protein to identify novel and effective drug candidates to combat HIV associated diseases. The ethical approval for this work was granted by Taif University on 27th July 2022 and IRB 43-808.

## Methods

This study is a computational structure-based drug design study, carried out at University of Taif, Taif, Saudi Arabia and African Genome Centre (AGC), Mohammed VI Polytechnic University, Benguerir, Morocco from January 2021 to March 2022. Before making the study design, we carried out a review of the existing literature using Google scholar. BMS-488043 inhibitor of HIV was selected to be used in this study among the reported approved inhibitors owing to the its viral susceptibility and pharmacokinetics profile.

### Preparation of protein

Viral protein gp120 3D structure under the accession code 3DNN was retrieved from Protein DataBank.^27^ Removal of water and energy minimization was performed followed by protein protonation by using MOE software

### Ligand preparation

Using chem-office software, the BMS-488043 compound was sketched and then saved as a mol extension for opening in MOE software. In MOE, the mol file of compound BMS-488043 was protonated and energy minimization was done. It was then saved in mdb file for docking purpose. Molecular docking: The active residues for gp120 viral protein were selected from previous literature. Then to predict bioactive conformations of ligand (BMS-488043), the ligand was docked with gp120 viral protein using the MOE-Dock program such as the Triangular Matching docking method. The ligand marvelously fits into the active site by mediating hydrogen bonding interactions with gp120 active site including His105, Trp112, Asp113, Lys121, Asp368, Ser375, Gln422, Met426, Gln428, Lys429, Arg476.

### Prediction of protein stability

The gp120/BMS-488043, a protein-ligand complex, was investigated for its stability and domain dynamics, using molecular dynamics (MD) simulation. Molecular dynamics simulations provide structural information on ligands, receptors interaction, and biomolecules. To solvate the system, the TIP3P water model was utilized. Further, to neutralize the system, the counter ions were added. For electrostatic interaction Particle Mesh Ewald algorithm was utilized. For constraining the bonds, SHAKE algorithm was utilized and finally, 100ns MD simulation was carried out.

### Systematic analysis

Analysis was done in Amber v2018. Pharmacophore modeling and validation: The minimized conformer of the gp120/BMS-488043 was used for complex pharmacophore generation. The 3D pharmacophore building was carried out in MOE from (gp120/BMS-488043) complex. From binding interactions, vital features were known that were detected in (gp120/BMS-488043) complex for building pharmacophore. Finally, it was validated via a test database. Test data set was screened and mapped on the complex-based pharmacophore model consists of 7 features.

### Pharmacophore-based screening

Virtual screening was performed for identifying lead compounds. In this connection, the ChemBridge (2020, San Diego, CA, USA) and ZINC (2015, San Francisco, CA, USA) database were utilized for pharmacophore-based screening. The obtained compounds were further filter out by using RO5 as drug properties can be identified by RO5.

### Interaction study

All retrieved hits were docked with gp120 viral protein to identify interaction and lead compounds. For every ligand, 20 confirmations were obtained. Poses from Cambridge and ZINC database were selected for further screening. Through LigPlot, binding interaction and proteins were visualized. All compounds were carried out for MD simulation. The binding mode, interaction, binding affinity and visual inspection demonstrated that selected compounds may be serve as potent structurally diverse inhibitors of HIV-1.

## Results

### Construction of pharmacophore

**Figure 1:**
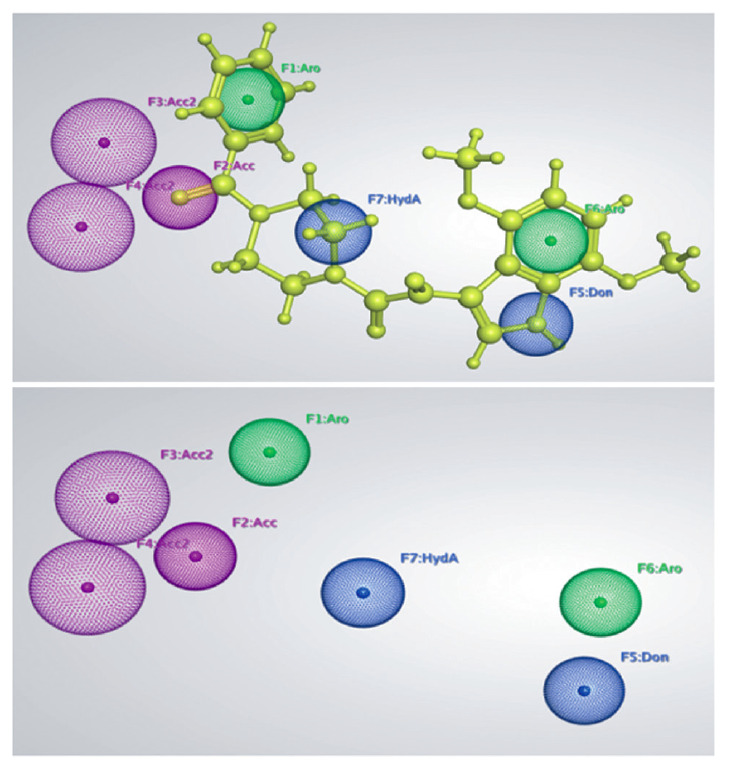
- A 3D structure-based pharmacophore features generated by the knowledge of molecular interactions between gp120 and BMS-488043. The H-bond acceptor features are shown in purple color, Aromatic features are shown in green color while H-bond donor and hydrophobic features are shown in blue color. BMS-488043 mapped on the pharmacophore features are shown in yellow color.

A fascinating property of the pharmacophoric model is the recognition of contact sites, which can contribute to improved selectivity and affinity for binding. The MOE sfotware was used to create a gp120 and macrocyclic inhibitor (BMS-488043) complex-based pharmacophore model. LigPlot, a MOE plugin, was used to define the essential chemical features for generating pharmacophore models based on molecular binding interactions gp120 and BMS-488043 docked complex. Three hydrogen bond acceptors (Acc), one hydrophobic feature (HydA), one hydrogen bond donor (Don), and 2 aromatic rings were the main features of the resulting pharmacophore model. Figure 1 showed the generated pharmacophore model in which aromatic features (F1 and F6) were shown in green, hydrogen bond acceptor features (F2, F3, F4) were shown in purple, and donor and hydrophobic features (F5 and F7, respectively) were shown in blue color. The oxygen atoms of the dimethoxy, benzoylpiperazin, and Ethan-1, 2-dione of the ligand act as H-bond acceptors (Acc), and demonstrated the contact with the His105 residue. Similarly, the nitrogen atom of the 2, 5-dimethoxypyridine and the hydrogen of the NH moiety of the ethylamine serve as H-bond donor and mediate interactions with the active residues Asp368 and Lys429. Five of the 7 features (F1, F2, F3, F4, and F5) were designated as important features. A test dataset of 7 known inhibitors was used to validate the created pharmacophore model. All the 7 known inhibitors were aligned on to the created pharmacophoric features and analyzed. It was interesting to note that the generated pharmacophore model picked the compounds of the test dataset with mapping all the 7 features.

### Virtual screening and binding energy

The pharmacophore-based virtual screening method is very important and useful, and it is extensively used in medicinal chemistry research projects, with the goal of facilitating the rapid discovery of drug-like, potent, and novel compounds.^28-30^ Potential compounds against gp120 were extracted from the ChemBridge and ZINC database via a 3D query based on the validated pharmacophore model. As a result of screening, 311 and 45 compounds were retrieved from ChemBridge and ZINC databases respectively and these hit compounds were found to be mapped with the pharmacophore features. According to Lipinski’s rule of 5 (RO5), drug-like compounds with log *p*-values below 5, log S values below 5, a molecular weight below 500 Da, a number of hydrogen bond acceptors below 10, and a number of hydrogen bond donors below 5 or else has poor permeation or absorption.^31^ The obtained hits from pharmacophore-based screening were shortlisted on the basis of RO5, as a result 150 and 42 hit compounds from ChemBridge and ZINC databases were obtained, respectively, and further subjected to molecular docking.

### Molecular docking

**Figure 2:**
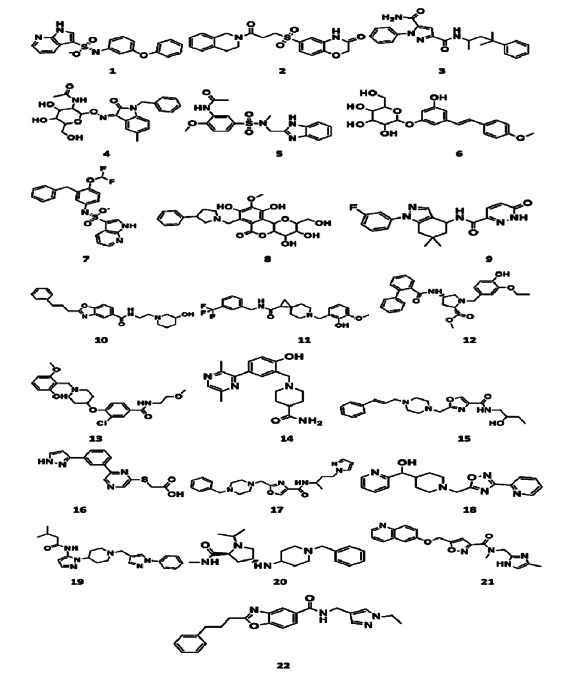
- Structures of 22 retrieved hits from ZINC and Cambridge database.

To determine the binding mode and affinity, the retrieved hits were docked into the gp120 binding site using the MOE docking protocol. Based on the docking score top-ranked 100 compounds from ChemBridge and 25 compounds from ZINC database were selected. These selected compounds were further evaluated by visualizing their binding interactions by using the LigX plot, implemented in the MOE software. The hit compounds that mediate substantial interactions with the important amino acid residues of the active site of gp120 including His105, Trp112, Asp113, Asp368, Asn425, Met426, Trp427, Gln428, Lys429, Gly431, Lys432, Gly473, Asp474 and Arg476 were considered as the potential virtual hits (ligands). Based on their interactions with the active residues of the gp120 binding pocket, 45 and 22 compounds from the ChemBridge and ZINC databases, respectively, were selected as hit compounds. These selected compounds were then further screened based on their binding affinity and binding energy (Table 1 and Figure 2).

**Table 1:** - Results of molecular docking studies and physiochemical properties of the hit compounds from Chembridge and ZINC database.

S. No	Compound ID	Docking score (S)	Binding energy (kcal/mol)	Binding Affinity (kcal/mol)	Physiochemical properties				
					Mol. weight	Log P	Donor	Acceptor	Log S
1	ZINC72027118	-7.43	-13.81	-5.66	364.405	4.74	1	4	-5.59
2	ZINC06893293	-9.65	-18.82	-7.24	400.455	2.03	1	5	-3.82
3	ZINC78871949	-9.56	-18.36	-7.05	390.487	3.45	2	3	-5.63
4	ZINC10271817	-9.76	-17.34	-7.30	469.494	0.43	4	7	-4.14
5	ZINC28082519	-8.26	-16.07	-6.56	388.448	2.61	2	5	-3.57
6	ZINC04543426	-8.59	-26.06	-7.94	404.415	0.75	2	5	-3.35
7	ZINC72014953	-9.05	-22.35	-6.72	428.439	5.14	1	5	-5.74
8	ZINC79193975	-11.12	-19.24	-8.12	487.505	1.15	5	9	-2.82
9	ChemBridge14695864	-9.21	-27.59	-7.83	381.411	2.27	2	4	-5.11
10	ChemBridge16586658	-8.75	-23.02	-8.12	407.514	3.19	2	4	-4.62
11	ChemBridge16610636	-9.30	-23.41	-7.73	448.485	5.10	2	4	-4.55
12	ChemBridge17115705	-11.05	-19.28	-7.27	474.557	4.27	2	5	-6.39
13	ChemBridge18573235	-10.08	-23.57	-8.05	448.947	3.74	2	6	-4.36
14	ChemBridge31483125	-8.18	-12.49	-5.49	340.427	2.43	2	5	-1.98
15	ChemBridge32351948	-7.86	-15.72	-5.58	398.507	2.27	2	5	-2.84
16	ChemBridge36158778	-7.41	-15.17	-7.44	312.353	2.71	2	5	-3.42
17	ChemBridge39229227	-8.35	-21.83	-6.28	422.533	3.19	1	5	-2.66
18	ChemBridge46500603	-8.53	-18.75	-5.99	351.410	2.83	1	6	-2.85
19	ChemBridge58982400	-7.47	-18.30	-6.42	401.551	2.63	2	5	-2.52
20	ChemBridge78620441	-8.25	-19.03	-6.00	386.492	1.46	4	6	-2.41
21	ChemBridge80485459	-8.07	-17.54	-5.40	405.502	3.69	1	5	-2.84
22	ChemBridge83081330	-7.96	-21.91	-8.06	383.488	4.05	1	4	-3.69

**Figure 3:**
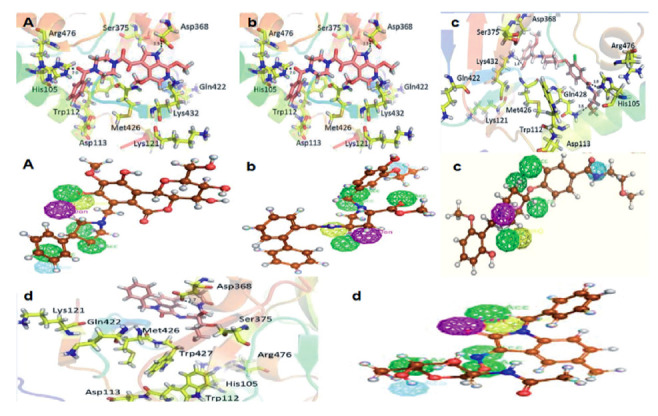
- Pharmacophore mapping and binding interactions of selected compounds. The upper panel of A), b), c) and d) showed the binding interactions of compound 8,12,13, and 4, respectively. The lower panel of A), b), c) and d) showed the pharmacophore mapping of compound 8,12,13, and 4, respectively.

In the present research investigation, finally, 4 compounds were selected on the basis of significant binding affinity and interactions. The hit compounds numbered 4, 8, 12, and 13 in Table 1 were obtained as most promising virtual hits owing to their significant interactions with all the crucial residues of gp120 as shown in Figure 3.

### Pan assay interference compounds (PAINS) filter assay

It is suggested that pharmacokinetic properties of the compounds be studied early on in the process of drug design and development in order to reduce the risk of late failure in clinical development. The PAINS are false positives that interact with several biological targets. Thus PAINS serve as an electronic filter, to reduce the false positives. The hits obtained from docking studies were passed through PAINS filter (https://www.cbligand.org/PAINS/search_struct.php). Table 2 represents the compounds that successfully passed the PAINS filter.

**Table 2:** - Structure and Simplified Molecular-Input Line-Entry System (SMILE) ID of compounds that successfully passes the pan assay interference compounds filter.

Ligand	PAINS Filter	Structure	SMILE ID
ChemBridge14695864	Passed	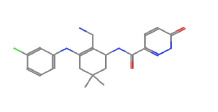	c1(cccc(c1)NC1=C(C(CC(C1)(C)C)NC(=O)c1n[nH]c(=O)cc1)CN)F
ChemBridge16586858	Passed	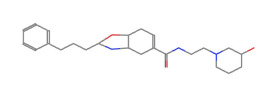	c1ccc(cc1)CCCC1NC2C(O1)CC=C(C2)C(=O)NCCN1CCCC(C1)O
ChemBridge16610636	Passed	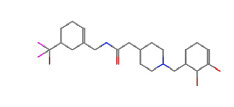	C1C(CC(=CC1)CNC(=O)CC1CCN(CC1)CC1C(C(=CCC1)[O-])O)C(P)(I)P
ZINC06893293	Passed	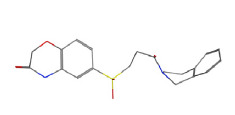	O=C(CCS(=O)(=O)c1ccc2c(c1)NC(=O)CO2)N1CCc2ccccc2C1

### Molecular dynamics simulation

To understand the binding mechanism of BMS-488043, ChemBridge14695864, and ZINC06893293, a docking simulation was implemented which directed the ligands into the active site of gp120 protein. The docking analysis predicted that BMS-488043 establishes 2 hydrogen bond contacts with the binding site residues Asp368 and Arg476. Considering dynamic properties is essential for understanding certain aspects of protein function. Furthermore, molecular docking does not account for the effect of solvent. Therefore, MD simulation was carried out to determine the dynamic behavior of protein-ligand complexes in an explicit water solvent, to investigate the receptor conformational change induced by BMS-488043, ChemBridge14695864, and ZINC06893293.

**Figure 4:**
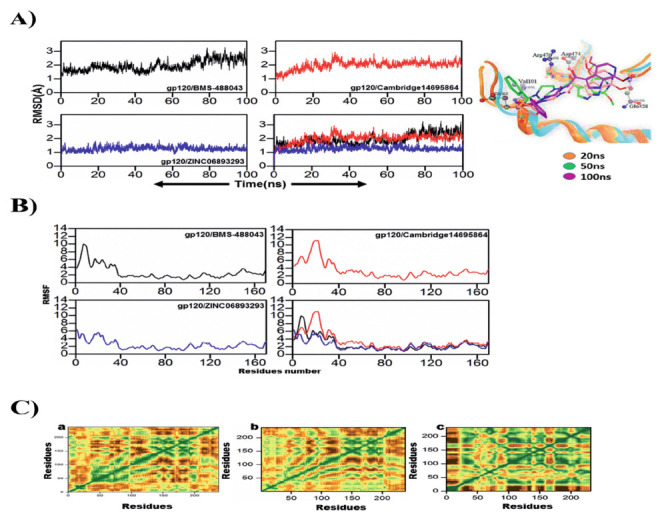
- Dynamic analysis of simulated complexes. A) RMSD of BMS-488043, Cambridge14695864 and ZINC06893293 in complex with gp120 during the 100ns of simulation. B) RMSF of gp120 residues after binding of BMS-488043, Cambridge14695864 and ZINC06893293 during the 100ns of simulation. C) Dynamics cross‐correlation matrix of (a) gp120/BMS-488043, (b) gp120/Cambridge14695864, and (c) gp120/ZINC06893293.

Figure 4A suggested that gp120 in complex with ChemBridge14695864 and ZINC06893293 showed more stable root mean square deviation (RMSD) as evaluate the gp120/ BMS-488043 complex. All the 3 systems showed RMSD of less than 3Å. Glycoprotein120/BMS-488043 complex fluctuate between 1.5 to 3Å with variable fluctuations till the end of the simulation. Initially, gp120/ChemBridge14695864 complex showed fluctuation however, after 50ns the RMSD become stable which suggested that the complex attained its stability. In case of gp120/ZINC06893293 most stable RMSD of less than 2Å was observed with inconsiderable fluctuations throughout the simulation of 100ns.

Root mean square fluctuation (RMSF) was computed to analyze the ligand-induced flexibility/rigidity in the amino acid residues of gp120 (Figure 4B). The plot suggested that all the systems were showed similar pattern of fluctuations however, the magnitude was different. The residues 40-170 in gp120/ZINC06893293 and gp120/ChemBridge14695864 showed lesser fluctuation than the gp120/ BMS-488043 complex but the residues 0-40 showed higher fluctuation in gp120/ChemBridge14695864 as compared to other 2 systems. The complex gp120/ZINC06893293 shows overall less fluctuation throughout 100ns simulation which reflect stable mode of inhibition of ZINC06893293. Decreased fluctuation in the residues of the binding site of gp120 upon binding of ZINC06893293 suggests that probability of further associations in the region might be relatively lowered. This strengthen the gp120/ZINC06893293 capability of becoming a potential inhibitor of gp120.

### Displacements of functional residues analysis

Using dynamics cross-correlation matrices (DCCM), the residual fluctuation in all 3 complexes were determined. In each of the 3 complexes, we analyzed the patterns of both positively and negatively correlated motions. The investigation revealed that each of the 3 complexes exhibited substantial patterns of corelated motions. Figure 4C (a) for gp120/BMS-488043 complex explains that interactions are slightly increased the positive correlation in the binding site residues 60-70 and 140-170. This showed stable binding of gp120/BMS-488043 in the active site and that it’s binding at the site may distinctly affect the stability of other residues that are crucial in binding. Figure 4C (b) for gp120/ChemBridge14695864 explains that binding in the active site reflects a slight decreased in positive correlation in the binding site residues as compared to gp120/ChemBridge14695864 however, slight increase in negetive correlation was also observed.

As evident from Figure 4C (c), gp120/ZINC06893293 displayed overall random correlation. A deep analysis showed that gp120/ZINC06893293 binding displayed some distinct positive correlations at numerous positions suggesting that the constancy of the important residues may depend on the interactions with the ligand. In general, all of the complexes displayed a favourable positive correlation, most prominently in the binding region, whereas the negative correlations were seen in the residues of the non-binding site. These DCCM results are also consistent with trends from the RMSD and RMSF for all the 3 complexes.

## Discussion

Human immunodeficiency virus’ gp120 glycoprotein bound to the host cell’s CD4 receptor, allowing the virus to enter the cell. Targeting gp120-CD4 protein-protein interaction is an attractive strategy to inhibit the virus entry. Substantial progress have been made to develop HIV entry inhibitors and several potential inhibitors have been developed. BMS-488043, an indole derivative is reported to inhibit HIV entry by targeting gp120-CD4 interaction. The molecular interactions between gp120 and BMS-488043 is valuable for further development of new inhibitors. Herein, we generated a valid structure-based pharmacophore model by using the details of molecular interactions between gp120 and BMS-488043 for the identification of potential HIV inhibitors. In contrast to ligand-based pharmacophore model, which need ligands in their bioactive conformation, structure-based pharmacophore model do not require ligand information. It is based on the interations between the protein and the ligands. The advantage of structure-based over ligand-based pharmacophore model is that it is less biased towards existing ligand chemotypes and permit the identification of novel scaffolds.^32^

The final validated pharmacophore model which consists of 7 crucial features was used to virtually screen the small molecule library of ChemBridge and ZINC database. The compounds with the significant fitness with the pharmacophore model were further subjected to molecular docking studies. We delineated that the selected compound would inhibit gp120 binding to the CD4 receptor in a manner similar to BMS-488043 by analysing the molecular interactions between the compounds and the gp120. Moreover, to evaluate the cross-reactivities of the obtained hit compounds, pan assay interface compounds were predicted. The pan assay interface compounds are those compounds that non-specially react with the many biological targets and ultimately cause toxicity and off-target side effects. It was interesting to note that the selected compounds passed the PAINS filter.

For a more in-depth analysis of the selected virtual hits’ dynamic behaviour in relation to the gp120-BMS-488043 complex, MD simulation was performed. The results of MD simulation reveal that the 2 selected compounds namely ChemBridge14695864 and ZINC06893293 were significantly stabilized the gp120 protein in comparison of BMS-488043 as shown by the RMSD and RMSF plots. Taken together, the multistep virtual screening strategy resulted in the identification of 2 diverse chemical compounds as potential virtual hits against HIV. The binding interactions and dynamic stability of the 2 selected compounds indicated that these compounds might show similar potency like BMS-488043, however, further experimental validation is needed.

### Study limitations

Few limitations to this study were identified. The compounds were identified only on the basis of pharmacophore model’s defined chemical features of BMS-488043 in complex with gp120, which can cause issues if the model contains more or fewer features than the actual substrate. Moreover, structural modifications to proteins that are critical to their function typically occur on time scales of the nanosecond to the microsecond. Therefore, 100 nanosecond of MD simulation is too short to explore actual conformational changes. However, gp120/ZINC06893293 and gp120/ChemBridge14695864 complexes were converged after 50ns and remain stable till the end of the simulation. In this study, computational methods suggested 2 diverse inhibitors of HIV-1 however, experimental validation is needed to further validate the potencies of these compounds.

In conclusion, the virus HIV causes acquired immune deficiency syndrome and the literature suggested that there are approximately 35.3 million HIV-positive people worldwide. In spite of the availability of several HIV therapies, there is an immediate need for effective anti-HIV medications or vaccine to combat the global HIV epidemic. In this study, we successfully identified the 2 compounds with the different chemical scaffold against HIV-1 gp120 by constructing structure-based pharmacophore model in combination with molecular docking and MD simulation. The identified hit compounds exhibited strong binding interactions and significant stability during the molecular docking and MD simulation studies, respectively. It would be interesting to further performed *in vitro* and *in vivo* studies to validate the theoretical results.

## References

[1] Baucom C , Bate J , Ochoa S , Santos I , Sergios A , Lorentzen L , et al. The epidemiology of the aids pandemic: historical, cultural, political, societal perspectives and knowledge of HIV. J Stud Res 2019; 8.

[2] Eisinger RW , Fauci AS. Ending the HIV/AIDS pandemic^1^ . Emerg Infect Dis 2018; 24: 413–416. 29460740 10.3201/eid2403.171797 PMC5823353

[3] Bruxelle J-F , Trattnig N , Mureithi MW , Landais E , Pantophlet R. HIV-1 entry and prospects for protecting against infection. Microorganisms 2021; 9: 228. 33499233 10.3390/microorganisms9020228 PMC7911371

[4] Stockdale AJ , Saunders MJ , Boyd MA , Bonnett LJ , Johnston V , Wandeler G , et al. Effectiveness of protease inhibitor/nucleos(t)ide reverse transcriptase inhibitor–based second-line antiretroviral therapy for the treatment of human immunodeficiency virus type 1 infection in sub-Saharan Africa: A systematic review and meta-analysis. Clinical Infectious Diseases 2018; 66: 1846–1857. 29272346 10.1093/cid/cix1108 PMC5982734

[5] Chen GJ , Sun HY , Chang SY , Cheng A , Huang YS , Lin KY , et al. Effectiveness of switching from protease inhibitors to dolutegravir in combination with nucleoside reverse transcriptase inhibitors as maintenance antiretroviral therapy among HIV-positive patients. Int J Antimicrob Agents 2019; 54: 35–42. 30905695 10.1016/j.ijantimicag.2019.03.016

[6] Obasa AE , Mikasi SG , Brado D , Cloete R , Singh K , Neogi U , et al. Drug resistance mutations against protease, reverse transcriptase and integrase inhibitors in people living with HIV-1 receiving boosted protease inhibitors in South Africa. Front Microbiol 2020; 11: 438. 32265875 10.3389/fmicb.2020.00438 PMC7099763

[7] Chimukangara B , Lessells RJ , Sartorius B , Gounder L , Manyana S , Pillay M , et al. HIV-1 drug resistance in adults and adolescents on protease inhibitor-based antiretroviral therapy in KwaZulu-Natal province, South Africa. J Glob Antimicrob Resist 2022; 29: 468–475. 34785393 10.1016/j.jgar.2021.10.023

[8] de la Peña AT , Sanders RW. Stabilizing HIV-1 envelope glycoprotein trimers to induce neutralizing antibodies. Retrovirology 2018; 15: 63. 30208933 10.1186/s12977-018-0445-y PMC6134781

[9] Beitari S , Wang Y , Liu SL , Liang C. HIV-1 Envelope glycoprotein at the interface of host restriction and virus evasion. Viruses 2019; 11: 311. 30935048 10.3390/v11040311 PMC6521621

[10] Lu W , Chen S , Yu J , Behrens R , Wiggins J , Sherer N , et al. The polar region of the HIV-1 envelope protein determines viral fusion and infectivity by stabilizing the gp120-gp41 association. J Virol 2019; 93: e02128–e02218. 30651369 10.1128/JVI.02128-18 PMC6430531

[11] Prévost J , Tolbert WD , Medjahed H , Sherburn RT , Madani N , Zoubchenok D , et al. The HIV-1 Env gp120 Inner Domain Shapes the Phe43 Cavity and the CD4 Binding Site. mBio 2020; 11: e00280–e00320. 32457241 10.1128/mBio.00280-20 PMC7251204

[12] Rullo EV , Ceccarelli M , Condorelli F , Facciolà A , Visalli G , D’Aleo F , et al. Investigational drugs in HIV: Pros and cons of entry and fusion inhibitors (Review). Mol Med Rep 2019; 19: 1987–1995. 30628713 10.3892/mmr.2019.9840

[13] Allen AG , Chung C-H , Atkins A , Dampier W , Khalili K , Nonnemacher MR , et al. Gene editing of HIV-1 co-receptors to prevent and/or cure virus infection. Front Microbiol 2018; 9. 30619107 10.3389/fmicb.2018.02940 PMC6304358

[14] Smith LK , Babcock IW , Minamide LS , Shaw AE , Bamburg JR , Kuhn TB. Direct interaction of HIV gp120 with neuronal CXCR4 and CCR5 receptors induces cofilin-actin rod pathology via a cellular prion protein- and NOX-dependent mechanism. PLoS One 2021; 16: e0248309. 33705493 10.1371/journal.pone.0248309 PMC7951892

[15] Smith LK , Kuhn TB , Chen J , Bamburg JR. HIV associated neurodegenerative disorders: a new perspective on the role of lipid rafts in gp120-mediated neurotoxicity. Current HIV Research 2018; 16: 258–269. 30280668 10.2174/1570162X16666181003144740 PMC6398609

[16] Nickoloff-Bybel EA , Festa L , Meucci O , Gaskill PJ. Co-receptor signaling in the pathogenesis of neuroHIV. Retrovirology 2021; 18: 24. 34429135 10.1186/s12977-021-00569-x PMC8385912

[17] Lahiry P , Torkamani A , Schork NJ , Hegele RA. Kinase mutations in human disease: interpreting genotype–phenotype relationships. Nat Rev Genet 2010; 11: 60–74. 20019687 10.1038/nrg2707

[18] Lai YT. Small Molecule HIV-1 Attachment inhibitors: discovery, mode of action and structural basis of inhibition. Viruses 2021; 13: 843. 34066522 10.3390/v13050843 PMC8148533

[19] Meuser ME , Murphy MB , Rashad AA , Cocklin S. Kinetic characterization of novel HIV-1 entry inhibitors: discovery of a relationship between off-rate and potency. Molecules 2018; 23: 1940. 30081466 10.3390/molecules23081940 PMC6222832

[20] Zhao C , Princiotto A , Farrell M , Smith III AB, Madani N, Sodroski J. Investigating the mechanism of a unique human immunodeficiency virus-1 (HIV-1) entry inhibitor, MF275. Open Forum Infect Dis 2018; 5: S200–S201.

[21] Hosny A , Ashton M , Gong Y , McGarry K. The development of a predictive model to identify potential HIV-1 attachment inhibitors. Comput Biol Med 2020; 120: 103743. 32421648 10.1016/j.compbiomed.2020.103743

[22] Mostashari Rad T , Saghaie L , Fassihi A. HIV-1 entry inhibitors: a review of experimental and computational studies. Chem Biodivers 2018; 15: e1800159. 30027572 10.1002/cbdv.201800159

[23] Rodríguez-Izquierdo I , Natalia C , García F , los Ángeles Muñoz-Fernandez M de. G2-S16 sulfonate dendrimer as new therapy for treatment failure in HIV-1 entry inhibitors. Nanomedicine 2019; 14: 1095–1107. 31066644 10.2217/nnm-2018-0364

[24] Yin S , Zhang X , Lai F , Liang T , Wen J , Lin W et al. Trilobatin as an HIV-1 entry inhibitor targeting the HIV-1 Gp41 envelope. FEBS Lett 2018; 592: 2361–2377. 29802645 10.1002/1873-3468.13113

[25] Curreli F , Belov DS , Kwon YD , Ramesh R , Furimsky AM , O’Loughlin K et al. Structure-based lead optimization to improve antiviral potency and ADMET properties of phenyl-1H-pyrrole-carboxamide entry inhibitors targeted to HIV-1 gp120. Eur J Med Chem 2018; 154: 367–391. 29860061 10.1016/j.ejmech.2018.04.062 PMC5993640

[26] Wang T , Ueda Y , Zhang Z , Yin Z , Matiskella J , Pearce BC , et al. Discovery of the human immunodeficiency virus type 1 (HIV-1) attachment inhibitor temsavir and its phosphonooxymethyl prodrug fostemsavir. J Med Chem 2018; 61: 6308–6327. 29920093 10.1021/acs.jmedchem.8b00759

[27] Liu J , Bartesaghi A , Borgnia MJ , Sapiro G , Subramaniam S. Molecular architecture of native HIV-1 gp120 trimers. Nature 2008; 455: 109–113. 18668044 10.1038/nature07159 PMC2610422

[28] Sabe VT , Ntombela T , Jhamba LA , Maguire GEM , Govender T , Naicker T , et al. Current trends in computer aided drug design and a highlight of drugs discovered via computational techniques: A review. Eur J Med Chem 2021; 224: 113705. 34303871 10.1016/j.ejmech.2021.113705

[29] da Silva Rocha SFL , Olanda CG , Fokoue HH , Sant’Anna CMR. Virtual screening techniques in drug discovery: review and recent applications. Curr Top Med Chem 2019; 19: 1751–1767. 31418662 10.2174/1568026619666190816101948

[30] Williams JC , Opare S , Sugadoss SK , Ganesan A , Kalyaanamoorthy S. Chapter 5 - Virtual screening techniques in pharmaceutical research. In: Török B , Dransfield T , Török M (eds). Contemporary Chemical Approaches for Green and Sustainable Drugs. Elsevier; (Amsterdam) Netherlands: 2022. pp 89–128.

[31] Karami TK , Hailu S , Feng S , Graham R , Gukasyan HJ. Eyes on Lipinski’s rule of five: a new “Rule of Thumb” for physicochemical design space of ophthalmic drugs. JJ Ocul Pharmacol Ther 2022; 38: 43–55. 10.1089/jop.2021.0069 PMC8817695 34905402

[32] A. Sanders MP , McGuire R , Roumen L , Esch IJP de , Vlieg J de , G. Klomp JP , et al. From the protein’s perspective: the benefits and challenges of protein structure-based pharmacophore modeling. MedChemComm 2012; 3: 28–38.

